# The Midwifery services framework: What is it, and why is it needed?

**DOI:** 10.1016/j.midw.2017.11.003

**Published:** 2018-02

**Authors:** Andrea Nove, Petra ten Hoope-Bender, Nester T. Moyo, Martha Bokosi

**Affiliations:** a Novametrics Ltd, 4 Cornhill Close, Duffield, Derbyshire, DE56 4HQ, United Kingdom; b UNFPA, Switzerland; c International Confederation of Midwives, The Netherlands

**Keywords:** AAAQ, availability, accessibility, acceptability, quality, ERA, education, regulation, association, HRH, human resources for health, ICM, International Confederation of Midwives, MDG, millennium development goal, MMR, maternal mortality ratio (maternal deaths per 100,000 live births), MSF, Midwifery Services Framework, PMNCH, Partnership for Maternal, Newborn and Child Health, SDG, sustainable development goal, SRHR, sexual and reproductive health and rights, SRMNH, sexual, reproductive, maternal and newborn health, TWG, technical working group, UNFPA, United Nations Population Fund, WHO, World Health Organisation, Midwifery, Health workforce, Sexual, reproductive, maternal and newborn health, Human resources for health, Sustainable development goals, Universal health coverage

## Abstract

Most low- and middle-income countries failed to meet the Millennium Development Goal targets for maternal, newborn and child health, and even more ambitious targets have been set under the Sustainable Development Goals and the Ending Preventable Maternal Mortality initiative. This means that many countries will need to accelerate progress on sexual, reproductive, maternal and newborn health over the next few years. Recent years have seen the publication of a large and convincing body of evidence about the potential of midwifery to make a significant contribution to this acceleration, but little practical guidance has emerged to help countries invest in midwifery services so that their health systems can meet the increasing need for sexual, reproductive, maternal and newborn health care. To help fill this gap, the International Confederation of Midwives designed and launched the Midwifery Services Framework, a new tool to guide countries through the process of strengthening and developing their midwifery services. This first of a series of three papers introduces the MSF, explains why it is needed, how it was developed, its guiding principles and its anticipated outcomes and impact. The other two papers explain the process of implementing the Midwifery Services Framework, and lessons learned in the first countries to start implementation.

## Highlights

• Acceleration is needed in progress on sexual, reproductive, maternal&newborn health.
• Investing in the midwifery workforce is an effective strategy for improving outcomes.
• The Midwifery Services Framework (MSF) provides guidance on how to do this.
• This paper introduces the MSF, explains why it is needed and how it was developed.
• It is a modular tool, applicable in all contexts.

## Introduction

Despite significant progress since 2000, most low- and middle-income countries failed to meet the 2015 targets set under the Millennium Development Goals relating to maternal and child health (Requejo et al., 2015). It is estimated that there were over 300,000 maternal deaths in the world in 2015, and nearly all of the preventable deaths occurred in low- and middle-income countries (WHO et al., 2015). In addition, 2.7 million neonatal deaths and 2.6 million stillbirths occurred across the world in 2015 (Healthy Newborn Network, 2016, UN Inter-agency group for child mortality estimation, 2015 ), mostly in low- and middle-income countries.

Launched in 2015, the sustainable development goals (SDGs) set ambitious targets for universal coverage of essential health services, including sexual and reproductive health and rights (SRHR) (United Nations, 2016). These targets include reducing the global maternal mortality ratio (MMR) to fewer than 70 maternal deaths per 100,000 live births by 2030. To achieve this target, the global MMR will need to reduce by at least 7.5% each year between 2016 and 2030: a huge acceleration on the 2.3% annual rate of reduction observed between 1990 and 2015 (WHO et al., 2015). There are also national targets under the Ending Preventable Maternal Mortality initiative: by 2030, every country should reduce its MMR by two-thirds from the 2010 baseline, and by 2030 no country should have an MMR higher than 140 (World Health Organization, 2015). The SDG targets also include the ending of preventable deaths of newborns and children under 5 years of age, with all countries aiming to reduce neonatal mortality to at least as low as 12 neonatal deaths per 1000 live births and under-5 mortality to at least as low as 25 deaths per 1000 live births.

These ambitious mortality targets will be achieved only with universal access to high quality sexual, reproductive, maternal and newborn health (SRMNH) care services, including for family planning, information and education, and the integration of reproductive health into national strategies and programmes. In turn, this will require substantial increases to health financing and the recruitment, development, deployment and retention of the health workforce, especially in developing countries. This was underlined in the 2015 Global Strategy for Women's, Children's and Adolescents’ Health (Every Woman Every Child, 2015), which stated that the health workforce – including midwives – is ‘a critical area for investment’. In 2016 the World Health Assembly adopted *Workforce 2030: the Global Strategy on human resources for health* (HRH) (World Health Organization, 2016a). This strategy has the overall goal ‘to improve health, social and economic development outcomes by ensuring universal availability, accessibility, acceptability and quality of the health workforce’. In 2017, the World Health Organisation (WHO) launched the Quality of Care Network, with the aim of achieving significant reductions in maternal and newborn mortality via improvements to quality of care. The monitoring framework for this initiative counts the health workforce as one of five key inputs to the process (World Health Organization, 2017a).

Midwives are an important element of the health workforce; there is a wealth of recent, high-quality evidence demonstrating the impact and effectiveness of midwifery in improving SRMNH outcomes. The 2014 State of the World's Midwifery report concluded that midwives, when educated and regulated to international standards, can meet 87% of the global need for essential SRMNH services (UNFPA et al., 2014). There is also evidence indicating that investment in midwives and midwifery is a cost-effective approach to the reduction of maternal and neonatal mortality and stillbirth (Homer et al., 2014), and that midwifery-led models of care result in excellent maternal and neonatal outcomes (Sandall et al., 2016).

On the basis of this body of evidence, midwives are seen as fundamental to the provision of quality care for women and newborns (Koblinsky et al., 2016). In 2016, the Director General of the WHO urged ‘… midwives to continue to make a difference through the provision of high-impact and low-cost interventions. Strengthening nursing and midwifery to support universal health coverage is a key imperative for improving the health of populations’ (World Health Organization, 2016b). Similarly, the United Nations Population Fund (UNFPA) is prioritising investment in the midwifery workforce to help achieve its SRHR goals: its current strategic plan includes a target for the number of countries in which the development of midwifery workforce policies is based on current global standards (United Nations Population Fund, 2014).

Yet there remains a massive global shortage of midwives (UNFPA et al., 2014), and recent research indicates that those midwives who are in the workforce often feel disrespected and undervalued in the workplace and/or the community, which limits their ability to meet the needs of women and newborns (International Confederation of Midwives et al., 2016). There is also evidence that midwives are not valued professionally due to the socio-cultural feminisation of midwifery, which has led to under-investment in midwifery education, regulation and services (Filby et al., 2016). There is, therefore, a demonstrable need for strengthening of the midwifery profession and work environment to enable midwives to make the necessary contribution to development goals and targets.

At a global level, the International Confederation of Midwives (ICM) plays a central role in strengthening the profession, based on its mission ‘to strengthen Midwives Associations and to advance the profession of midwifery globally by promoting autonomous midwives as the most appropriate caregivers for childbearing women and in keeping the birth normal, in order to enhance the reproductive health of women, and the health of their newborn and their families’ (International Confederation of Midwives, 2014). In the context of this overall mission, ICM's strategic and business plans are organised around five strategic objectives: (1) strengthen midwifery education, continuing education programmes and the role of the midwife as an educator, (2) enhance midwives’ professional autonomy and ensure midwifery regulation, education and practice is designed and governed by midwives, (3) promote midwifery research that enhances and documents evidence-based midwifery practice, (4) advocate for midwifery and extend the influence of midwives in policy development that drives service direction, and (5) pursue strategic collaborations with relevant organisations and networks that share a common interest. ICM therefore plays a central linking role between the global dialogues and initiatives described above and the delivery of effective midwifery services ‘on the ground’, by working with national professional associations and other stakeholders to strengthen midwifery services and improve the quality of midwifery care.

As part of this role, ICM has recently designed the Midwifery Services Framework (MSF) (International Confederation of Midwives, 2015) and has supported eight countries to begin implementation. This paper is the first of a series of three articles about the MSF; it aims to introduce the MSF, explain its objectives and describe how it was developed. The second paper details the process of the MSF implementation in the countries so far involved, and the third describes the lessons learned to date from the first countries to begin implementation.

## What is the MSF and how is it distinct from other initiatives and tools?

A framework can be defined as a structure, overview or outline containing a number of descriptive categories (such as concepts or variables) and the relations between them, that are assumed to explain or account for an observed phenomenon (Sabatier, 2007). The MSF is a framework which aims to structure the process of developing and strengthening the midwifery profession to the extent that it is competent and enabled to meet the SRMNH needs of women and newborns and thus contribute to improving SRMNH outcomes. It is a tool to assist countries to operationalise this process, and is designed to be applicable to all national health systems regardless of whether or not the country already has a cadre of health worker called ‘midwife’. This is because the practice of midwifery as defined in the Lancet Series on Midwifery exists in all countries: ‘… skilled, knowledgeable, and compassionate care for childbearing women, newborn infants, and families across the continuum throughout pre-pregnancy, pregnancy, birth, post-partum, and the early weeks of life’ (Renfrew et al., 2014).

The MSF is based on the premise that midwives are the most appropriate health workers to deliver midwifery care according to the Lancet definition, assuming that they are educated according to global standards (International Confederation of Midwives, 2013a) and can work within an enabling environment. This is because core characteristics of midwifery include: ‘optimising normal biological, psychological, social, and cultural processes of reproduction and early life; timely prevention and management of complications; consultation with and referral to other services; respect for women's individual circumstances and views; and working in partnership with women to strengthen women's own capabilities to care for themselves and their families’ (Renfrew et al., 2014). Midwives’ unique role across the whole continuum of SRMNH care makes them particularly well-placed to demonstrate these characteristics (UNFPA et al., 2014).

The MSF aligns with, but is distinct from, other tools such as the *H4+ Handbook for conducting a workforce assessment* (UNFPA and WHO, 2015) and the WHO *Strengthening Midwifery Toolkit* (World Health Organization, 2011). The WHO *Toolkit* sets out the standards that should be met by the midwifery profession (including the ICM global standards); the MSF is a practical tool to support countries to work towards achieving those standards. Like the *H4+ Handbook*, the MSF takes the needs of women and newborns as its basis and includes a robust assessment of SRMNH worker availability, accessibility, acceptability and quality (AAAQ), but the MSF has several additional stages which support national stakeholders to work together to identify and address the challenges and bottlenecks affecting the AAAQ of the workforce. MSF implementation also includes support to strengthen national midwifery professional associations, which has been recognised as an effective strategy for addressing HRH issues such as skills gaps and ‘brain drain’ (McQuide et al., 2007).

## Objectives and principles of the MSF

The overarching objective of the MSF is to provide practical guidance and supporting tools that can be used by health care decision-makers in any country to initiate, develop, strengthen, monitor and/or evaluate midwifery services (depending on how well-developed these services are in that country) such that they can meet the needs of the population. Within this overall objective, the MSF aims to deepen understanding of the fundamental role that midwifery care plays in improving women's and children's health, and to build on the current global commitment to reducing maternal, newborn and child mortality and ill-health with a practical approach to making midwives accessible to families.

The MSF is designed to align with the global architecture described in the Introduction, and also the Global Investment Framework for Women's and Children's Health (Stenberg et al., 2014), thus supporting countries to achieve global and national targets for improving SRMNH outcomes. For example, the MSF addresses all the objectives of the Global Strategy on HRH (World Health Organization, 2016a): it aims to optimise workforce performance via effective policy-making, it is needs-based, it helps to build country capacity for evidence-based policy-making and it helps to ensure accountability for implementing national strategies. It is designed to be a living document which is updated regularly to include new evidence, tools and approaches, as well as lessons learned from country implementation.

The process is led by the national government and the midwifery profession in the implementing country, but involvement from other key stakeholders such as the ministries of education, finance and planning as well as civil society groups is advised so that all relevant parties feel ownership of the process and its outcomes.

## How was the MSF developed?

The process of establishing the MSF was informed by all the ‘interventions’ midwives do to maintain physiology and address pathology (Renfrew et al., 2014). The conceptual framework underpinning the development of the MSF was ICM's three-pillar approach to strengthening midwifery: education, regulation and strengthening professional associations, known by the acronym ‘ERA’ (International Confederation of Midwives, 2017). To operationalise this framework, an evidence-based, pragmatic and country focused programme was needed, that could address the country-specific issues that impact on the quality and accessibility of midwifery services. In other words, the process as a whole would need to go beyond just ERA, because midwives function with and within a broader health system and are part of the overall national health workforce (Campbell et al., 2015).

**Fig. 1:**
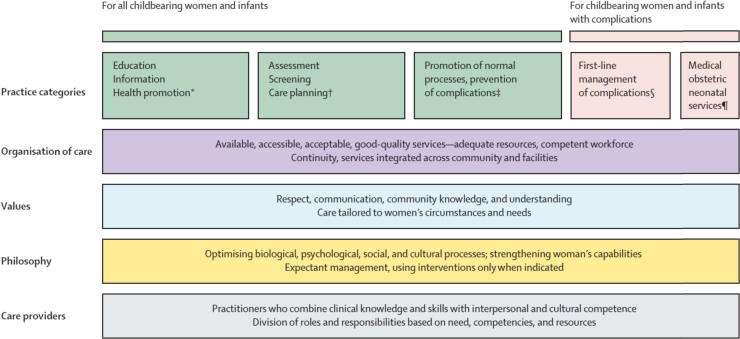
The Quality maternal newborn care framework.

*Source*: (Renfrew et al., 2014).

Other frameworks were therefore considered to supplement and complement the ERA framework: the Framework for Quality Maternal Newborn Care (Fig. 1) that was developed for the Lancet Series on Midwifery (The Lancet, 2014), and the AAAQ framework as it applies to the health system and health workforce (UNFPA et al., 2014). Both of these frameworks begin with a consideration of what women and their families need, indicating that the MSF needed to start from this basis. For this reason, the first two steps of the MSF focus on what women and families need and what the health system should be capable of to secure the fulfilment of these needs.

The process of developing the MSF was a collaborative one, involving discussions between ICM and its strategic partners, including UNFPA's sexual and reproductive health branch and WHO's health systems and health workforce departments. It was also informed by a literature review on the topics of: models of care, quality standards for obstetric care, health system strengthening, health worker deployment and retention strategies, and continuous professional development.

The design and content of the MSF was informed by data and evidence from a wide range of sources, including: the PMNCH Effective Interventions for SRMNH (The Partnership for Maternal Newborn and Child Health, 2011), the Lancet Series on Midwifery 2014 (The Lancet, 2014), both State of the World's Midwifery reports (UNFPA, 2011, UNFPA et al., 2014), global standards for education and regulation (International Confederation of Midwives, 2013b, International Confederation of Midwives, 2011a), the OneHealth tool (World Health Organization, 2017b), the Human Development Index (United Nations Development Programme, 2017), global data repositories such as the WHO Global Health Observatory and World Bank Open Data (World Bank, 2017, World Health Organization, 2017c), WHO health workforce data and tools (World Health Organization, 2014), the WHO ‘strengthening midwifery’ toolkit (World Health Organization, 2011), and national applications of ICM's Member Association Capacity Assessment Tool (International Confederation of Midwives, 2011b).

## Brief description of the MSF

The MSF process is described in detail in the second paper of this series, but in summary, it guides countries through a systematic process of identifying how well the midwifery workforce is meeting the country's SRMNH needs, and what needs to happen to address any identified gaps in provision. The process begins with meetings between key stakeholders, then a workshop attended by representatives of government ministries, civil society organisations, development partners and health worker professional associations. The outcome of this workshop is a clear list of issues to be addressed and the resources required to improve SRMNH services. The next step is to prioritise the list and sort the items on it into groups according to (1) their timeframe (short-, medium- and long-term) and (2) the resourcing implications (issues that can be solved without additional resources, those that can be delivered using domestic resources, and those for which additional funds and/or external expertise need to be sourced). Based on this list, Technical Working Groups (TWGs) are set up to address the identified issues, and a national steering committee is initiated, with a remit to monitor and supervise the activities of the TWGs. Each TWG develops a budgeted strategic plan complete with timelines of when they will report to the steering committee. Monitoring and evaluation systems are built in from the start.

The MSF methodology ensures that the whole process is tailored to the country context, and that there is government leadership in the planning and management of the activities carried out by the TWGs. ICM remains available to provide technical support for a period of two years after the initial workshop, e.g. if an issue is identified which requires expertise not available within the country, ICM will identify appropriate experts from its global network to support the country to address that issue.

**Fig. 2:**
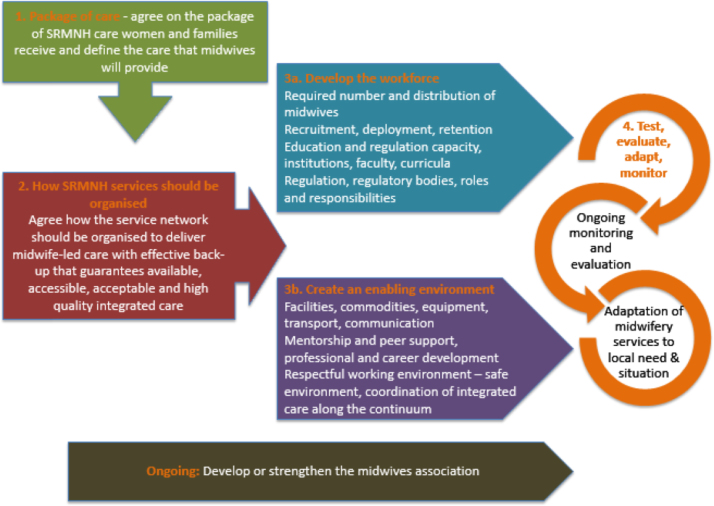
Midwifery Services Framework flowchart.

The MSF contains a set of service and workforce development modules (Fig. 2) that can be used in sequence or individually as appropriate for the country context. Each module builds on the previous one, with development or strengthening of the national midwifery association running in parallel to the modular activities.

The first module requires the country to consider which SRMNH services their citizens should be entitled to receive. The second module facilitates decisions about how services should be organised and delivered. The third module is split into two: (a) how the workforce needs to be developed, and (b) what needs to happen to the working environment, so that health workers can deliver the full range of SRMNH care and services. These are treated as two halves of the same module because in many countries the two processes will be led by different groups of stakeholders, but they should be undertaken concurrently to ensure co-ordination between the workforce and the work environment. The fourth and final module is the start of an ongoing process of making sure that the activities undertaken under the MSF are achieving the desired results.

## Anticipated outcomes of MSF implementation

The MSF was first launched in three countries: Lesotho, Afghanistan and Kyrgyzstan. Since then, the process has been initiated in an additional five countries: Bangladesh, Ghana, Togo, Uganda and Zimbabwe. The process is ongoing in all eight of these early adopting countries, so it is too early to provide a comprehensive assessment of the initiative's outcomes and impacts. It is, however, anticipated that successful implementation of the MSF will lead to a number of outcomes, including:

• A broader sense of ownership of and responsibility for the delivery of SRMNH services, due to the inter-sectoral and multistakeholder nature of the process
• Services being shaped around the needs of women and their families, due to use of data and evidence, and to the involvement of women and families in the process
• Improved sustainability of improvements to SRMNH services due to monitoring and evaluation being built in from the outset
• Improved country capacity to meet global and national objectives and targets for SRMNH outcomes
• Improved gender equity and women's empowerment (as the midwifery workforce and user base are predominantly female)
• Stronger professional associations and greater visibility and recognition of the midwifery profession due to its pivotal role in the process and the support provided by ICM

## Conclusion

The MSF was developed in response to evidence of (a) the potential of midwifery to make a significant contribution to universal health coverage and sustainable development, and (b) a historical lack of investment in midwifery in many contexts. A structured and evidence-informed process was needed to support countries to address these issues and thus to make better progress towards universal coverage of SRMNH services and targets set under global architecture such as the SDGs and the Global Strategy on HRH. This initial series of three papers is designed to introduce this new initiative and share information about lessons learned within the first 2 years of implementation, to help countries decide whether this type of process would be helpful and feasible for their context. A more detailed evaluation of the outcomes and impacts of the MSF is planned for when the early implementing countries have progressed further with the process.

## References

[bib1] Campbell J., Cometto G., Rasanathan K. (2015). Improving the resilience and workforce of health systems for women's, children's, and adolescents' health. BMJ.

[bib2] Every Woman Every Child, 2015. The global strategy for women’s, children’s and adolescents' health (2016–2030). Every Woman Every Child, New York.

[bib3] Filby A., McConville F., Portela A. (2016). What prevents quality midwifery care? A systematic mapping of barriers in low and middle income countries from the provider perspective. PLoS One.

[bib4] Healthy Newborn Network, 2016. Numbers [WWW Document]. URL 〈http://www.healthynewbornnetwork.org/numbers/〉 (Accessed 21 October 2016).

[bib5] Homer C.S.E., Friberg I.K., Dias M.A.B. (2014). The projected effect of scaling up midwifery. Lancet.

[bib6] International Confederation of Midwives, 2017. Education, regulation and association [WWW Document]. URL 〈http://internationalmidwives.org/what-we-do/education-regulation-association/〉 (Accessed 27 July 2017).

[bib7] International Confederation of Midwives, 2015. The ICM Midwifery Services Framework for Reproductive, Maternal, Neonatal, Child Health Services [WWW Document]. URL 〈http://www.internationalmidwives.org/projects-programmes/icm-msf-page1/〉 (Accessed 29 July 2017).

[bib8] International Confederation of Midwives (2014). Strategic Directions 2014–2017: A Promising Future.

[bib9] International Confederation of Midwives, 2013a. Essential Competencies for Basic Midwifery Practice [WWW Document]. URL 〈http://internationalmidwives.org/what-we-do/education-coredocuments/essential-competencies-basic-midwifery-practice/〉 (Accessed 26 June 2017).

[bib10] International Confederation of Midwives, 2013b. Global standards for midwifery education (2010): Amended 2013 [WWW Document]. URL 〈http://internationalmidwives.org/assets/uploads/documents/CoreDocuments/ICMStandardsGuidelines_ammended2013.pdf (Accessed 29 July 2017). 10.1016/j.midw.2011.04.001 21550149

[bib11] International Confederation of Midwives, 2011a. Global standards for midwifery regulation [WWW Document]. URL 〈http://internationalmidwives.org/assets/uploads/documents/GlobalStandardsComptenciesTools/English/GLOBALSTANDARDSFORMIDWIFERYREGULATIONENG.pdf〉 (Accessed 29 July 2017).

[bib12] International Confederation of Midwives, 2011b. ICM member association capacity assessment tool (MACAT) and guidelines [WWW Document]. URL 〈http://internationalmidwives.org/what-we-do/association-coredocuments.html〉 (Accessed 27 July 2017).

[bib13] International Confederation of Midwives, World Health Organization, White Ribbon Alliance (2016). Midwives' Voices, Midwives' Realities: Findings from a Global Consultation on Providing Quality Midwifery Care.

[bib14] Koblinsky M., Moyer C.A., Calvert C. (2016). Quality Maternity Care For Every Woman, Everywhere: a Call to Action. Lancet.

[bib15] McQuide, P., Millonzi, K., Farrell, C., 2007. Strengthening health professional associations: Technical brief #8 [WWW Document]. URL 〈https://www.intrahealth.org/resources/strengthening-health-professional-associations〉 (Accessed 22 August 2017).

[bib16] Renfrew M.J., McFadden A., Bastos M.H. (2014). Midwifery and quality care: findings from a new evidence-informed framework for maternal and newborn care. Lancet.

[bib17] Requejo J., Victora C., Bryce J. (2015). A Decade of Tracking Progress for Maternal, Newborn and Child Survival: the 2015 Report.

[bib18] Sabatier P.A. (2007). Theories of the Policy Process.

[bib19] Sandall J., Soltani H., Gates S., Shennan A., Devane D. (2016). Midwifery-led continuity models versus other models of care for childbearing women. Cochrane Database Syst Rev.

[bib20] Stenberg K., Axelson H., Sheehan P. (2014). Advancing social and economic development by investing in women's and children's health: a new Global Investment Framework. Lancet.

[bib21] The Lancet, 2014. The Lancet Series on Midwifery [WWW Document]. URL 〈http://www.thelancet.com/series/midwifery〉 (Accessed 20 June 2017).

[bib22] The Partnership for Maternal Newborn&Child Health (2011). Essential Interventions, Commodities and Guidelines for Reproductive, Maternal, Newborn and Child Health.

[bib23] UN Inter-agency group for child mortality estimation (2015). Levels and Trends in Child Mortality Report 2015.

[bib24] UNFPA (2011). The State of the World's Midwifery 2011: Delivering Health, Saving Lives.

[bib25] UNFPA, WHO (2015). Conducting a Sexual, Reproductive, Maternal, Newborn And Adolescent Health Workforce Assessment.

[bib26] UNFPA, WHO, ICM (2014). The State of the World's Midwifery 2014: a Universal Pathway. A Woman's Right to Health.

[bib27] United Nations, 2016. Sustainable development goals [WWW Document]. URL 〈http://www.un.org/sustainabledevelopment/sustainable-development-goals/〉 (Accessed 21 November 2016).

[bib28] United Nations Development Programme, 2017. Human Development Index (HDI) [WWW Document]. URL 〈http://hdr.undp.org/en/content/human-development-index-hdi〉 (Accessed 17 August 2017).

[bib29] United Nations Population Fund (2014). UNFPA Strategic Plan 2014–2017.

[bib30] WHO, UNICEF, UNFPA, World Bank, UN Population Division (2015). Trends in Maternal Mortality: 1990 to 2015.

[bib31] World Bank, 2017. World Bank Open Data [WWW Document]. URL 〈http://data.worldbank.org/〉 (Accessed 17 august 2017).

[bib32] World Health Organization, 2017a. A network for improving quality of care for maternal, newborn and child health: monitoring framework [WWW Document]. URL 〈http://www.who.int/maternal_child_adolescent/topics/quality-of-care/quality-of-care-brief-m-e.pdf?Ua=1〉 (Accessed 24 August 2017).

[bib33] World Health Organization, 2017b. OneHealth tool [WWW Document]. URL 〈http://www.who.int/choice/onehealthtool/en/〉 (Accessed 17 August 2017).

[bib34] World Health Organization, 2017c. Global Health Observatory data respository [WWW Document]. URL 〈http://apps.who.int/gho/data/node.home〉 (Accessed 17 August 2017).

[bib35] World Health Organization (2016). Global Strategy on Human Resources For Health: Workforce 2030.

[bib36] World Health Organization (2016). Global Strategic Directions for Strengthening Nursing and Midwifery 2016–2020.

[bib37] World Health Organization (2015). Strategies Toward Ending Preventable Maternal Mortality (EPMM).

[bib38] World Health Organization, 2014. WHO Global Health Workforce statistics: 2014 update [WWW Document]. URL 〈http://www.who.int/hrh/statistics/hwfstats/en/〉.

[bib39] World Health Organization, 2011. Strengthening midwifery toolkit [WWW Document]. URL 〈http://www.who.int/maternal_child_adolescent/documents/strenthening_midwifery_toolkit/en/〉 (Accessed 27 July 2017).

